# A Case of Early Stage Bladder Carcinosarcoma in Late Recurrence of Urothelial Carcinoma after Transurethral Resection

**DOI:** 10.1155/2018/1405108

**Published:** 2018-01-15

**Authors:** Daisaku Hirano, Toshiyuki Yoshida, Daigo Funakoshi, Fuminori Sakurai, Shou Ohno, Yoshiaki Kusumi

**Affiliations:** ^1^Department of Urology, Higashimatsuyama Municipal Hospital, Higashimatsuyama 355-0041, Japan; ^2^Department of Urology, Nihon University School of Medicine, Tokyo 173-8610, Japan; ^3^Department of Urology, Kawaguchi Municipal Medical Center, Kawaguchi 333-0833, Japan; ^4^Department of Pathology, Nihon University School of Medicine, Tokyo 173-8610, Japan

## Abstract

Carcinosarcomas of the urinary bladder are rare biphasic neoplasms, consisting of both malignant epithelial and malignant mesenchymal components, and the prognosis of this tumor is unfavorable in most patients with even possibility of resection of disease. A 77-year-old male with a history of transurethral resection (TUR) of urothelial carcinoma (UC) of the bladder and adjuvant intravesical chemotherapy with pirarubicin 10 years ago revisited our department with a gross hematuria. Cystoscopy demonstrated an approximately 2.5 cm nonpapillary tumor on the right wall of the bladder. Pelvic MRI showed the tumor without extending the base of the bladder wall. The tumor could be completely removed with TUR. The malignant epithelial elements consisted of high-grade UC and the majority of mesenchymal components were fibrosarcomatous differentiation based on immunohistochemical studies. The tumor could be pathologically also suspected to be an early stage on TUR specimens. Although he has received no additional intervention due to the occurrence of myocardial infarction at three weeks after the TUR, he has been alive with no evidence of recurrence of the disease 27 months after the TUR. Some early stages of bladder carcinosarcoma might have a favorable prognosis without aggressive treatments.

## 1. Introduction

As to bladder cancers, urothelial carcinoma is histologically the most common (90%), followed by squamous cell carcinoma (5%) with mostly concomitant bladder stone, and less than 2% are adenocarcinoma or other variants [1]. Carcinosarcoma in the other variants is an extremely rare neoplasm of the urinary bladder, accounting for 0.1 to 0.3% of all bladder malignancies [2, 3]. Histologically, carcinosarcomas are defined by the World Health Organization as a biphasic tumor consisting of malignant epithelial and mesenchymal components [4]. The majority of carcinosarcomas are found at a more advanced stage at diagnosis and a poor prognosis in comparison with high-grade urothelial carcinoma [5]. Most patients with carcinosarcoma are treated with cancer-directed surgery such as transurethral resection and cystectomy with combined radiotherapy or chemotherapy [6]. However, no specific treatment guideline has been available for carcinosarcoma of the bladder because of the rarity of this tumor. Recently, we experienced a case of early stage bladder carcinosarcoma in late-onset recurrence after initial urothelial carcinoma treated with transurethral resection of bladder tumor (TURBT) and adjuvant intravesical chemotherapy for long-term.

## 2. Case Presentation

**Figure 1:**
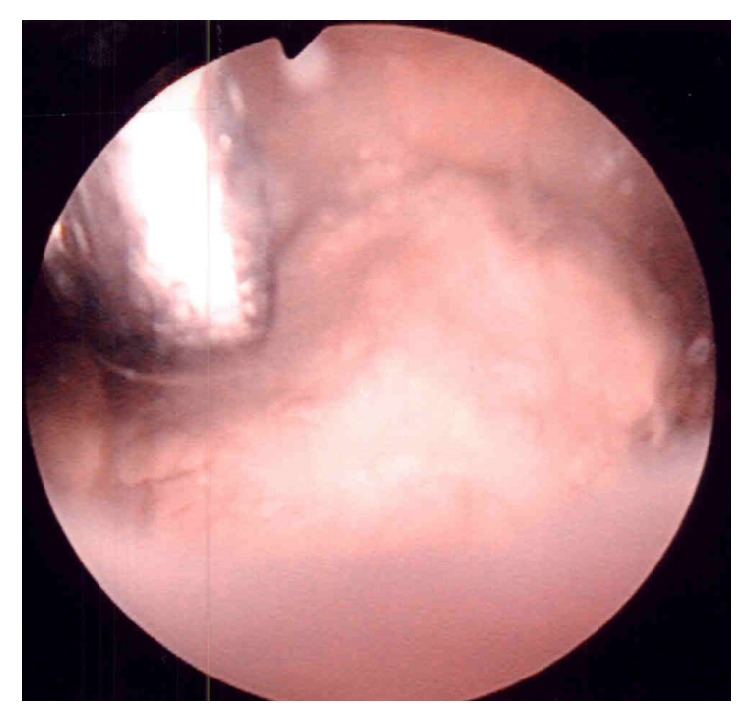
Endoscopic appearance of the tumor. Resectoscope reveals the tumor just before biopsy by forceps on TURBT.

**Figure 2:**
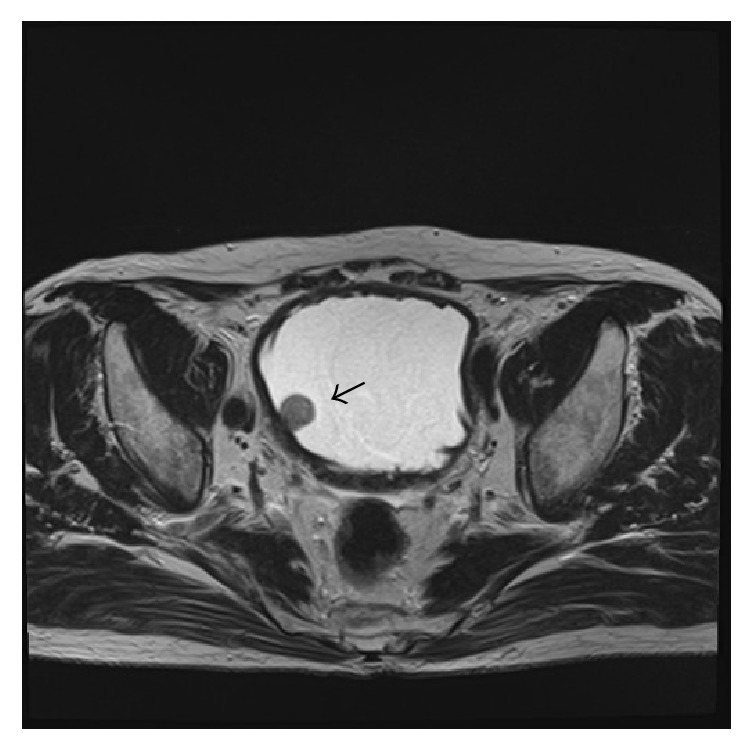
Pelvic MRI. Pelvic MRI T2 showed a 2.5 cm relatively homogenous mass (allow) in the right lateral bladder wall without extending base of the bladder wall.

A-77-year-old male was rereferred to our institution in April 2015 with a 2-week history of asymptomatic gross hematuria. He had no history of cigarette smoking. As to his illness survey, he had undergone TURBT for bladder cancer at our department 10 years ago. Histological examination of the TURBT specimens showed high-grade urothelial carcinoma with pT1. He received adjuvant intravesical chemotherapy with pirarubicin 20 mg every two weeks for approximately 3 years, with follow-up with cystoscopy every 3 to 6 months until April 2012. Thereafter, he have not visited our clinic. During this period, he had an onset of diabetes mellitus and cerebral infarction. Cystoscopy revealed nonpapillary bladder tumor on the right wall.  Figure 1 shows the tumor before resection during TURBT. Pelvic MRI T2 showed a 2.5 cm relatively homogenous mass in the right lateral bladder wall without extending the base of the bladder wall (Figure 2). Voided urine cytology appeared negative. There were no abnormalities in regular laboratory tests and the other imaging diagnosis. TURBT was performed with completely removing the tumor and specimens of the tumor base after the resection was sampled with punch biopsies.

**Figure 3:**
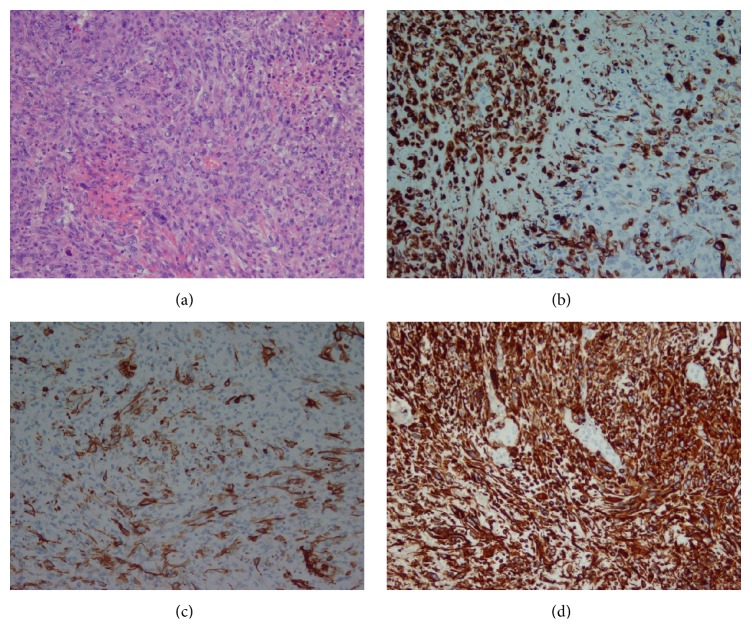
Histopathology of the tumor. (a) Hematoxylin-eosin stain. The epithelial part of the tumor consists of high-grade urothelial carcinoma and the mesenchymal part is composed of oval to spindle shaped cells proliferation. (b) Immunohistochemical staining indicates CK-7 positive. (c) Immunohistochemical staining shows  *α*-smooth muscle actin positive. (d) Immunohistochemical staining reveals vimentin positive.

Histopathological study of the resected urinary bladder tissues showed carcinosarcoma composed of biphasic malignant epithelial and mesenchymal cells. The epithelial part of the tumor consisted of high-grade urothelial carcinoma and the mesenchymal part composed of oval to spindle shaped cells proliferation (Figure 3(a)). Immunohistochemically, the malignant carcinomatous cells stained positive for cytokeratin 7 (CK-7) (Figure 3(b)). In contrast, the mesenchymal components stained positive for vimentin (Figure 3(c)) and  *α*-smooth muscle actin (*α*-SMA) (Figure 3(d)), but the other mesenchymal markers were negative.  Table 1 shows summary of immunohistochemical outcomes in the tumor. The malignant mesenchymal components could be considered to be fibrosarcomatous differentiation. The tumor base specimens obtained from the punch biopsies after the resection revealed no malignant cells in the muscle tissues. The pathological stage was pT1.

Although second-look TURBT was recommended, he eventually did not undergo it due to the occurrence of myocardial infarction at three weeks after the TURBT. He was treated with stents placement in the coronary artery with stents and has been continuing to take anticoagulant agents. He did not desire for additional intervention for the carcinosarcoma of the urinary bladder. After the TURBT, he has been followed with cystoscopy and urine cytology at every three months as well as imaging diagnosis such as chest-abdominal-pelvic computed tomography scan approximately every 3 to 6 months' interval. Fortunately, he has been alive with no evidence of recurrence of the disease 27 months after the TURBT.

## 3. Discussion

Carcinosarcoma of the urinary bladder has been reported as multiple terms such as sarcomatoid carcinoma, malignant mesodermal mixed tumor, spindle cell carcinoma, and giant cell carcinoma [7]. The histological characteristics of carcinosarcoma of the urinary bladder vary. In epithelial parts, most reported cases contain high-grade papillary/undifferentiated urothelial carcinoma, followed by small-cell carcinoma, squamous carcinoma, and adenocarcinoma, while in mesenchymal components the most common element is osteosarcoma followed by chondrosarcoma, rhabdomyosarcoma, leiomyosarcoma, liposarcoma, angiosarcoma, or multiple types of heterologous differentiation [8]. In this case, the mesenchymal elements composed of fibrosarcomatous malignant cells characterized by undifferentiated anaplastic spindle cells resembled mature fibroblast in a storiform pattern and the immunohistochemical analysis. As to the origin of urothelial carcinosarcoma, several investigators have suggested that these tumors might develop as a result of undifferentiated, totipotent neoplastic cells which undergo multiple pathways of terminal differentiation into either mesenchymal or epithelial elements [9]. In contrast, Gorstein and Anderson [10] have suggested that both malignant epithelial and mesenchymal components arise independently of each other based on immunohistochemical and electron microscopic features. Recent molecular genetic studies have shown support for a common monoclonal origin of both epithelial and mesenchymal component in carcinosarcoma of the urinary bladder [11, 12].

As to the etiology of carcinosarcoma of the urinary bladder, no definite risk factors have been identified to date; however, some investigators have suggested that it is associated with cigarette smoking like urothelial carcinoma [13]. Additionally, previous treatment with cyclophosphamide for previous conventional urothelial carcinoma in some patients and radiotherapy for previous endometrial carcinoma in a patient has been reported to result in sarcomatoid transformation [14]. Although the etiology of this case was not definitively identified, the long-term adjuvant intravesical therapy with pirarubicin for the previous urothelial carcinoma might be related to sarcomatoid transformation.

Hematuria has been the most common presenting symptom in patients with carcinosarcoma of the urinary bladder and next most frequently reported symptoms and signs of carcinosarcoma include dysuria, nocturia, acute urinary retention, lower abdominal pain, and urinary tract infection [7]. Several cases with a prior history of urothelial carcinoma were reported to be diagnosed by cystoscopy during regular follow-up [15]. Our case is, to our knowledge, the first reported case of late recurrence of urothelial carcinoma as the epithelial component of carcinosarcoma with an early stage. However, the majority of patients with carcinosarcoma have had high-histological grade and advanced disease at the time of presentation. Wang et al. [6] identified that 98.4% of 127 patients with urinary bladder carcinosarcoma showed poor or undifferentiated histology and 72.5% of 204 urinary bladder carcinosarcoma patients had regional or distant stage based on Surveillance, Epidemiology, and End Results (SEER) registry cases.

With respect to the treatment of carcinosarcoma of the urinary bladder, most patients with this disease have been treated with ultimately aggressive directed surgery such as radical cystectomy due to invasiveness of the tumor [7]. Nevertheless, the prognosis of this tumor is unfavorable in patients with even possibility of resection of disease. Several investigators [6] reported that the overall 5-year cancer-specific survival rate after cystectomy in their population study including 221 patients with carcinosarcoma of the urinary bladder was only 20.3%, suggesting a high risk of early dissemination, and identified that only tumor stage is an independent factor for disease-specific survival in multivariate analysis. Additionally, various combinations of neoadjuvant or adjuvant chemotherapy and/or radiotherapy after radical surgery have been advocated. Başeskioğlu et al. [13] retrospectively studied the efficacy of perioperative treatment with GC (cisplatin/gemcitabine) regimen in seven patients with sarcomatoid carcinoma of the urinary bladder, and this resulted in all patients dying within 48 months after surgery. The outcomes of even multimodal treatments are unsatisfactory. However, in some early stages aggressive treatments might be excessive as several investigators [16] reported a good outcome in a bladder carcinosarcoma without submucosal invasion removed with TURBT. In any case, multicentered, clinical trials are needed to establish a better therapeutic approach for this neoplasm.

In conclusion, carcinosarcoma of the urinary bladder is rare, aggressive, and lethal disease, and most patients with this disease have been treated with radical cystectomy and various combination therapies such as chemotherapy and radiotherapy. However, some carcinosarcomas with an early stage might have a favorable prognosis without aggressive treatments. Early detection is needed to improve the prognosis, and multicentric research is necessary to establish appropriate treatment recommendations for this malignancy.

## Figures and Tables

**Table 1:** Summary of immunohistochemical outcomes in the tumor.

Antibody	Staining
Cytokeratin 7	Positive
*α*-Smooth muscle actin	Positive
Desmin	Negative
Vimentin	Positive
Calponin	Negative
Myogenin	Negative
c-Kit	Negative
CD34	Negative
